# Repeated social defeat stress impairs attentional set shifting irrespective of social avoidance and increases female preference associated with heightened anxiety

**DOI:** 10.1038/s41598-018-28803-1

**Published:** 2018-07-11

**Authors:** Shu Higashida, Hirotaka Nagai, Kazuki Nakayama, Ryota Shinohara, Masayuki Taniguchi, Midori Nagai, Takatoshi Hikida, Satoshi Yawata, Yukio Ago, Shiho Kitaoka, Shuh Narumiya, Tomoyuki Furuyashiki

**Affiliations:** 1 Division of Pharmacology, Graduate School of Medicine, Kobe University, Kobe, 650-0017 Japan; 2 Laboratory for Advanced Brain Functions, Institute for Protein Research, Osaka University, Osaka, 565-0871 Japan; 3 Department of Biological Sciences, Graduate School of Medicine, Kyoto University, Kyoto, 606-8501 Japan; 4 Laboratory of Biopharmaceutics, Graduate School of Pharmaceutical Sciences, Osaka University, Osaka, 565-0871 Japan; 5 Medical Innovation Center, Graduate School of Medicine, Kyoto University, Kyoto, 606-8507 Japan; 6 Present Address: Department of Psychiatry, Yale University School of Medicine, New Haven, CT 06519 USA

## Abstract

Repeated social defeat stress (R-SDS) induces multiple behavioral changes in mice. However, the relationships between these behavioral changes were not fully understood. In the first experiment, to examine how the social avoidance is related to R-SDS-impaired behavioral flexibility, 10-week-old male C57BL/6N mice received R-SDS followed by the social interaction test and the attentional set shifting task. R-SDS impaired attentional set shifting irrespective of the development of social avoidance. In the second experiment, to examine whether R-SDS affects sexual preference and how this behavioral change is related to the social avoidance and R-SDS-heightened anxiety, another group of 10-week-old male C57BL/6N mice were subjected to R-SDS followed by the social interaction test, the female encounter test and the elevated plus maze test. The anxiety was heightened in the defeated mice without social avoidance, but not in those which showed social avoidance. Furthermore, female preference was increased specifically in the defeated mice which showed heightened anxiety, but was not related to the level of social avoidance. Together, these results showed that attentional set shifting is more sensitive to R-SDS than social interaction, and that female preference is affected by R-SDS in association with heightened anxiety rather than the social avoidance.

## Introduction

Repeated environmental stress induces behavioral changes, such as depression and heightened anxiety as well as impaired cognitive performance, in various animal species, and predisposes to mental illnesses in humans. To examine the effects of repeated environmental stress on multiple behaviors, various models of repeated or chronic stress, such as repeated social defeat stress (R-SDS), has been used. It has been shown that R-SDS induces decreased sucrose preference, social avoidance and heightened anxiety in mice. Since repeated treatment with antidepressants ameliorates some of these behaviors, namely decreased sucrose preference and social avoidance^1,2^, this stress model has been proposed to be a mouse model of depression^3–6^. It has been shown that the level of social avoidance varies among the defeated mice. Thus, the defeated mice which showed social avoidance or those which did not have been categorized as susceptible or resilient mice, respectively. Previous studies with this categorization method have reported the relationships between the social avoidance and other behavioral changes. For example, it has been reported that R-SDS decreased sucrose preference in susceptible, but not resilient, mice, whereas anxiety was heightened in both susceptible and resilient mice^7^.

Besides these behavioral changes, it has been shown that R-SDS as well as other types of repeated stress impairs behavioral flexibility as measured by attentional set shifting^8^. It remains to be examined how the social avoidance is related to R-SDS-impaired behavioral flexibility. Sexual preference is another stress-sensitive behavior: It has been shown that prolonged social isolation or corticosterone treatment in male mice decreases sexual preference to female mice^9^. Whether R-SDS affects sexual preference and how this behavioral change is related to the social avoidance and the heightened anxiety have not been examined, either.

In the present study, we examined the effects of R-SDS on attentional set shifting and female preference, and compared these effects with other R-SDS-induced behavioral changes. We performed two series of experiments. In the first experiment, mice were subjected to R-SDS followed by the social interaction test and the attentional set shifting task. We found that R-SDS impaired attentional set shifting in the defeated mice irrespective of the development of social avoidance. In the second experiment, mice were subjected to R-SDS followed by the social interaction test, the female encounter test and the elevated plus maze (EPM) test. We found that R-SDS heightened anxiety as measured by the EPM test in the defeated mice which did not develop social avoidance and that R-SDS increased female preference specifically in a subpopulation of defeated mice which showed heightened anxiety.

## Materials and Methods

### Animals

Nine-week-old male and female C57BL/6N mice and male ICR mice retired from breeding were purchased from Japan SLC (Shizuoka, Japan) and used for all the behavioral analyses, unless otherwise specified. We performed two series of behavioral experiments to examine the effects of R-SDS. The number of the mice used in the present study is summarized in Table 1. All the mice were maintained on a 12 h light/12 h dark cycle with food and water available ad libitum, except in the attentional set shifting task, in which mice were partially deprived of foods. All the mice were kept sexually naïve and did not receive any behavioral test until this study. All procedures for animal care and use were in accordance with the National Institutes of Health Guide for the Care and Use of Laboratory Animals and were approved by the Animal Care and Use Committees of Kobe University Graduate School of Medicine and Kyoto University Graduate School of Medicine.

**Table 1:** The number of mice used in the two series of experiments in the present study.

Behavioral flexibility	Ctrl	Defeat
· Repeated social defeat stress	10	11
· Social interaction test	10	11
· Visual cue task	10	11
· Response direction task	10	11
**Sexual preference and anxiety**	**Ctrl**	**Defeat**
· Repeated social defeat stress	32	63
· Social interaction test	32	63
· Female encounter test	32	63
· Elevated plus maze test	23	43

### Repeated social defeat stress

R-SDS was performed as previously described with minor modifications^10,11^. In this study, we performed R-SDS in two series of experiments, in one of which we tested behavioral flexibility using the attentional set shifting task after R-SDS and in another of which we tested sexual preference and anxiety using the female encounter test and the EPM test, respectively, after R-SDS. In the latter experiment, a subset of mice could not be subjected to the EPM test due to schedule constraints (see Table 1). In both experiments, we subjected 10-week-old male C57BL/6 N mice to R-SDS. We used only male mice because aggressor mice do not attack female mice if there is no genetic manipulation introduced in the aggressor mice^12^. Prior to R-SDS, male ICR mice were screened for their aggressiveness to a novel C57BL/6N mouse for 3 min daily for 3 days. We evaluated the aggression of the ICR mice by the latency and the number of attacks during the observation period, and only used those showing stable aggression for further experiments. Before R-SDS, male C57BL/6N mice were singly-housed with free access to food and water for a week. It has been reported that prior social isolation facilitated the development of depressive-like behavior due to R-SDS^13^, and that social isolation for a week (or up to three weeks) alone did not significantly induce depressive-like behavior or other behavioral changes in wild-type C57BL/6 mice^14^. These mice were then transferred to the home cage of a male ICR mouse for 10 min daily for 10 days. The pairs of defeated and aggressor mice were randomized daily to minimize the variability in the aggressiveness of aggressor mice. After the 10 min defeat episode, the mice were returned to their home cages and kept isolated until social defeat stress on the next day. Control mice were instead transferred to a novel cage and were allowed to freely explore for 10 min. We included all the data for the analyses without any exclusion.

### Social interaction test

**Figure 1:**
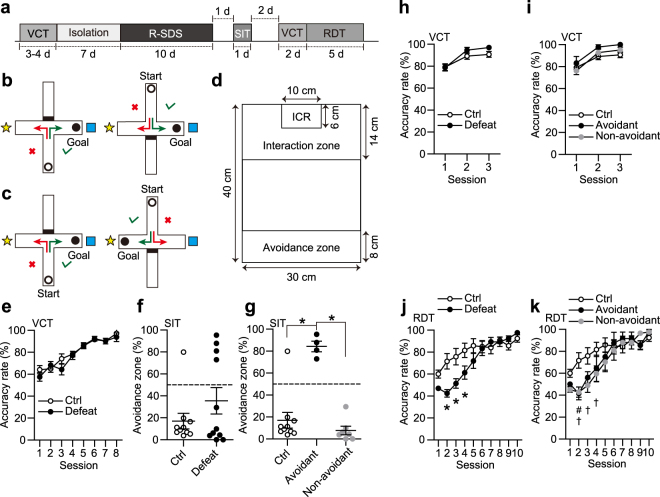
Repeated social defeat stress impaired the flexibility of learned behavior irrespective of the development of social avoidance. (**a**) A behavioral schedule. Mice received the visual cue task (VCT) for 3–4 days. Following social isolation for 7 days, the mice were subjected to R-SDS or cage transfers as control for 10 min daily for 10 consecutive days and received the social interaction test (SIT) after one-day interval. After the reminder trials of the VCT for 2 days, the defeated mice and the control mice received the response direction task (RDT) for 5 days. (**b**,**c**) Behavioral designs of the VCT (**b**) and the RDT (**c**). Blue boxes and yellow stars indicate visual cues. Mice were started from the Start location and made a turn to obtain a sucrose pellet located at the Goal location. See Materials and Methods for detail. (**d**) An experimental chamber used in the social interaction test (SIT). (**e**) Accuracy rates in the VCT of the mice to be defeated later or the control mice (“Ctrl”). (**f**–**k**) Time spent in the avoidance zone in the SIT (**f**,**g**), accuracy rates in the reminder trials of the VCT (**h,i**) and accuracy rates in the RDT (**j**,**k**) for defeated mice (“Defeat”) and control mice (“Ctrl”) (**f**,**h**,**j**) or for Avoidant mice and Non-avoidant mice (**g**,**i**,**k**). We categorized the defeated mice that spent more than 50% of the total time in the avoidance zone as the Avoidant mice and the others as the Non-avoidant mice. Values are expressed as means ± SEM. *P < 0.05 for one-way analysis of variance followed by multiple comparison tests with Tukey-Kramer correction (**g**) and for two-way repeated measures analysis of variance followed by multiple comparison tests with Sidak’s correction (**j**). (#, †)*P* < 0.05 for two-way repeated measures analysis of variance followed by multiple comparison tests with Tukey-Kramer correction between control mice and Avoidant mice (#) or Non-avoidant mice (†), respectively (**k**).

After R-SDS for 10 days, defeated mice and control mice were tested for their social interaction, as previously described^10,11^. Briefly, the mice were put in an open field chamber where a novel male ICR mouse was enclosed in a metal meshwork at one end (Fig. 1d), and the mice were allowed to freely explore the chamber for 150 s under video recording. All the mice were habituated to the same chamber in the absence of an ICR mouse for 150 s prior to the social interaction test. The areas in the chamber were divided into three; the interaction zone (closest to the ICR mouse), the middle zone and the avoidance zone (farthest to the ICR mouse). The time that each mouse spent in each zone was analysed post hoc using SMART video tracking software (PanLab Harvard Apparatus, Holliston, MA, USA).

### Attentional set shifting task

As described previously^15^, we performed the visual cue task and the response direction task to test the flexibility of associative learning in the defeated and control mice. We used the same apparatus in both of these tasks, but applied a different rule for a food reward. The apparatus contained four arms (25 cm × 5 cm, each of which had 15 cm-high transparent walls), and an opaque partition could be inserted at an entrance of each arm (see Fig. 1b,c). Visual cues, such as a ball and a cube, were placed around the apparatus. Prior to the visual cue task, male C57BL/6 N mice were deprived of foods for 36 h. Then, the amount of foods given was controlled throughout the task periods, so that the mice were maintained at 80% of their initial body weights. The mice were habituated to a sucrose pellet used as a food reward and also to the apparatus for 5 days. In the visual cue task, each of the mice was started from either the north or south arm. The opposite arm was closed with an opaque partition inserted at the entrance. The mouse had to learn to make a correct left or right turn according to the visual cues to obtain a sucrose pellet at the terminal of the fixed arm (see Fig. 1b). Each trial was ended, when the mouse reached at the end of either the left or right arm regardless of whether they reached the sucrose pellet. Mice received 12 trials per session and two sessions per day. Every mouse received no less than five sessions, until they achieved 11 or 12 successes in two consecutive sessions. Then the mice were subjected to R-SDS or its control procedures. Following R-SDS, all the mice received the visual cue task again as a reminder task to test the memory retention. Lastly, the mice received the response direction task. In this task, they were placed in the same apparatus as the visual cue task, but they had to make a turn to the same direction to obtain a sucrose pellet (see Fig. 1c). They received 12 trials per session and two sessions a day.

### Female encounter test

The female encounter test was performed as previously described with minor modifications^9^. Defeated and control mice of 13-week old at the test were placed in a center compartment of a behavioral chamber (42 cm × 50 cm × 30 cm, see Fig. 3a). This chamber was composed of three compartments separated by transparent partitions (30 cm × 30 cm). The mice were allowed to move freely between the compartments. Prior to the test, the mice were habituated to the behavioral chamber for 90 min. During the test, the mice were allowed to explore the chamber with novel male and female C57BL/6N mice of 9-week old as social targets for 10 min. A novel female mouse (either intact or ovarietomized) was enclosed in a mesh cage (10 cm × 6.5 cm × 20 cm) placed in a compartment on one end (“female zone”), and a novel male mouse was enclosed in another mesh cage placed in on another compartment on the other end (“male zone”). These two compartments were interconnected with the third compartment (“neutral zone”). The trajectory of the movement was recorded and analysed by SMART video tracking software. Female preference was calculated as the ratio of the time spent in the female zone to the time spent in either male or female zone.

### Elevated plus maze test

We performed the EPM test as previously described^11^. The apparatus consisted of two open arms (22.5 × 5 cm) and two closed arms (22.5 × 5 cm, attached with 15 cm-high gray walls) and was elevated at 50 cm from the ground. The test was conducted under white light at 5 lux. Mice were placed on an end of either of the closed arms and allowed to explore the maze for 5 min. The trajectory of the movement was recorded and analysed by the SMART video tracking software. The time spent in the open arms was used as an index for anxiety. Less time spent in the open arms is considered as a higher level of anxiety.

### Data availability statement

The datasets generated during and/or analysed during the current study are available from the corresponding author on reasonable request.

### Statistical analyses

Data are expressed as mean values ± SEM. Comparison of two groups was analysed by two-sided unpaired *t*-test. For comparison of more than two groups, one-way analysis of variance followed by multiple comparison tests with Tukey-Kramer correction or two-way repeated measures analysis of variance followed by multiple comparison tests with Sidak’s correction were used. The effect size of pairwise comparison was calculated by Hedge’s *g*^16^. For correlative analyses, a Pearson correlation was used. The analyses were performed with Prism 7.0 software (GraphPad). *P* values less than 0.05 were considered to be significant.

## Results

### R-SDS impaired attentional set shifting irrespective of the development of social avoidance

In the first experiment, we assessed the effects of R-SDS on behavioral flexibility, and compared it with R-SDS-induced social avoidance. For this purpose, we employed the attentional set shifting task comprising a visual cue task and a response direction task (Fig. 1a). In both of these tasks, a mouse was started from either the north or south arm and had to learn to make a correct turn to either of the remaining two arms to obtain a sucrose pellet. In the visual cue task, a sucrose pellet was located at the end of the same arm throughout the task (Fig. 1b), so that a mouse had to learn to make a correct turn according to visual cues. In the response direction task, a mouse had to learn to make a turn to the same direction, either left or right, to obtain a sucrose pellet throughout the task (Fig. 1c). Thus, the behavioral decision had to be made irrespective of visual cues. As the visual cue task was changed to the response direction task, a mouse had to change a behavioral strategy to obtain a reward. In this study, mice received the visual cue task and learned to make a correct turn beyond a certain accuracy (11 or 12 successes in 12 trials per session for two consecutive sessions) in 6–8 sessions (Fig. 1e). These mice were then subjected to either R-SDS or cage transfer as control. As previously reported^7,10^, approximately half of the defeated mice developed social avoidance, whereas other mice did not (Fig. 1d,f). For further analyses, we categorized the defeated mice that spent more than 50% of the total time in the avoidance zone as the Avoidant and the others as the Non-avoidant (Fig. 1g). The total time in the avoidance zone was significantly higher in the Avoidant mice than either the control (i.e. non-defeated) mice or the Non-avoidant mice, as one-way analysis of variance followed by Tukey-Kramer multiple comparisons test revealed a significant main effect of the Mouse group (*F*(2, 18) = 27.31, *P* < 0.0001) and statistically significant differences across the groups: control vs Avoidant (*P* < 0.0001, Hedge’s *g* = 3.33), Avoidant vs Non-avoidant (*P* < 0.0001, Hedge’s *g* = 7.67) and control vs Non-avoidant (*P* = 0.5334, Hedge’s *g* = 0.5). In reminder trials of the visual cue task after R-SDS, both the control mice and the defeated mice performed the task with high accuracy (Fig. 1h). The accuracy was similar irrespective of the development of social avoidance (Fig. 1i). These results suggest that the memory acquired in the visual cue task was well retained after R-SDS. In the response direction task, whereas both the control mice and the defeated mice learned to choose a correct arm eventually and reached the same plateau in six sessions, the accuracy of the defeated mice was significantly lower than that of the control mice in the second to fourth sessions (Fig. 1j). Two-way repeated measures analysis of variance followed by Sidak’s multiple comparisons test revealed a significant interaction between the Mouse group and the Session (*F*(9, 171) = 5.84, *P* < 0.0001) and a significant decrease in the performance of the defeated mice in the second (*P* = 0.0005, Hedge’s *g* = 1.68), third (*P* = 0.0068, Hedge’s *g* = 1.27) and fourth session (*P* = 0.0428, Hedge’s *g* = 1.00), as compared with the control mice. Thus, the defeated mice showed the delay in attentional set shifting from the previously acquired response in the visual cue task to the correct response in the response direction task, compared with the control mice. Unlike the social avoidance, attentional set shifting appears to be impaired similarly among the defeated mice. Indeed, both the Avoidant mice and the Non-avoidant mice showed similar delays from the control mice (Fig. 1k). Two-way repeated measures analysis of variance followed by Tukey-Kramer multiple comparisons test revealed a significant interaction between the Mouse group and the Session (*F*(18, 162) = 3.07, *P* < 0.0001) and a significant decrease in the performance of the Avoidant group in the second session (*P* = 0.0066, Hedge’s *g* = 1.49) and of the Non-avoidant group in the second (*P* = 0.0014, Hedge’s *g* = 1.52), third (*P* = 0.0029, Hedge’s *g* = 1.30) and fourth session (*P* = 0.0186, Hedge’s *g* = 1.11). These findings indicated that R-SDS impairs attentional set shifting irrespective of the development of social avoidance.

### R-SDS heightened anxiety in the defeated mice which did not show social avoidance

**Figure 2:**
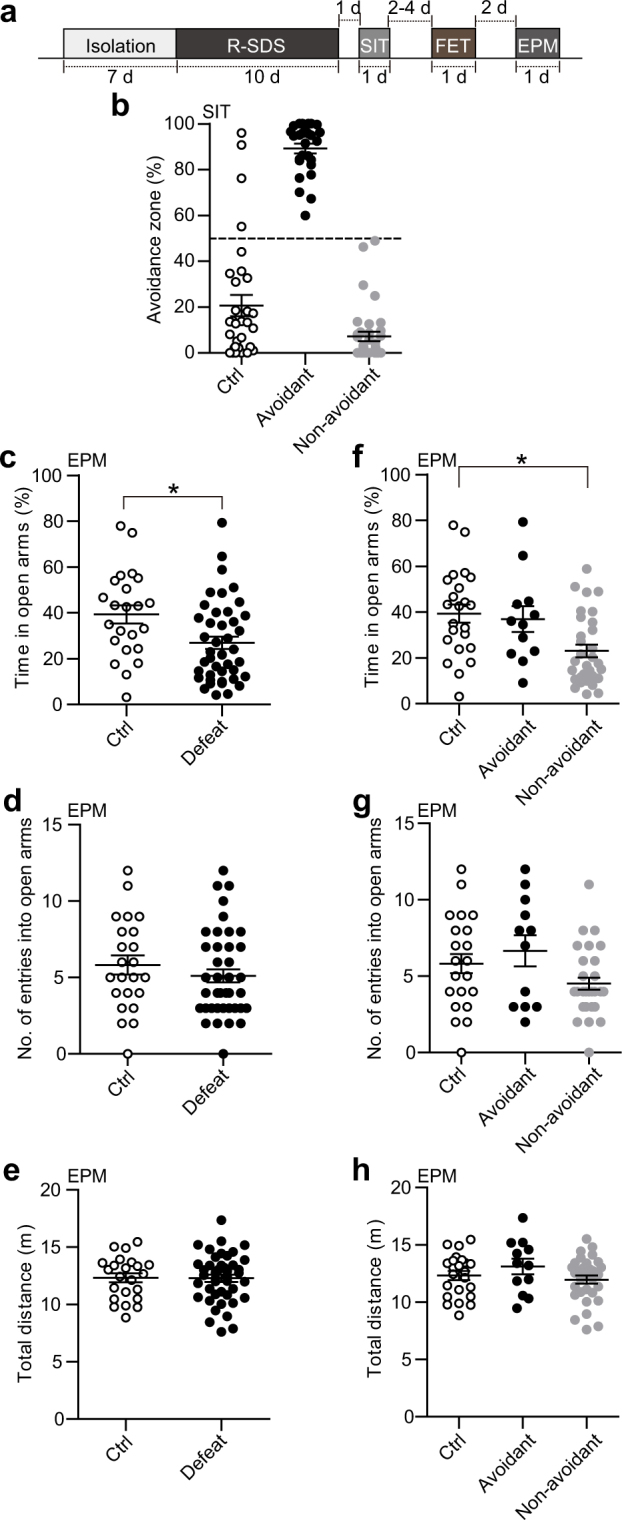
Repeated social defeat stress increased anxiety in the subpopulation of mice that didn’t show social avoidance. (**a**) A behavioral schedule. Following social isolation for a week, mice were subjected to R-SDS and cage transfers as control for 10 min daily for 10 consecutive days and received the social interaction test (SIT) for one day. Then the mice received the female encounter test (FET) followed by the elevated plus maze test (EPM). (**b**) Proportions of the time spent in the avoidance zone in the SIT for the control mice (“Ctrl”), Avoidant mice and Non-avoidant mice. We categorized the defeated mice that spent more than 50% of the total time in the avoidance zone as the Avoidant mice and the others as the Non-avoidant mice. (**c**–**h**) Proportions of the time spent in the open arms (**c**,**f**), numbers of entries to the open arms (**d**,**g**) and total distances moved (**e**,**h**) in the EPM for the control mice (“Ctrl”) and the defeated mice (“Defeat”) (**c**–**e**) or for the control mice (“Ctrl”), Avoidant mice and Non-avoidant mice (**f**–**h**). Values are expressed as means ± SEM. *P < 0.05 for unpaired *t* test (**c**) and one-way analysis of variance followed by multiple comparison tests with Tukey-Kramer correction (**f**).

In the second experiment, the effects of R-SDS on female preference, social interaction and anxiety-like behavior were assessed (Fig. 2a). As described in the first experiment, R-SDS-induced social avoidance varied among the defeated mice (Fig. 2b), and the Avoidant mice and the Non-avoidant mice as classified above were observed (27 and 36 mice, respectively), as similarly observed in the first experiment. R-SDS also decreased the time spent in the open arms in the EPM test, as reported previously^7^, indicating heightened anxiety (Fig. 2c; *t*(64) = 2.67, *P* = 0.0097, Hedge’s *g* = 0.69). Neither the number of entries to the open arms nor the locomotor activity in the EPM test were altered in the defeated mice, suggesting the lack of apparent locomotor deficits after R-SDS (Fig. 2d,e). The R-SDS-induced effects on the level of anxiety also appeared to vary among the defeated mice. Thus, we examined the level of anxiety in the Avoidant mice and the Non-avoidant mice (Fig. 2f). The time spent in the open arms was significantly decreased in the Non-avoidant, but not Avoidant, mice, relative to the control mice (Fig. 2f). One-way analysis of variance followed by Tukey-Kramer multiple comparisons test revealed a significant effect of the Mouse group (*F*(2, 63) = 6.56, *P* = 0.0026) and a significant decrease in the time spent in the open arms in the Non-avoidant mice compared to the control mice (*P* = 0.0033, Hedge’s *g* = 0.97). No significant difference was found in the locomotor activity among the three groups (Fig. 2g,h). These findings indicated that R-SDS heightened anxiety in the defeated mice which do not show social avoidance.

### R-SDS increased female preference in the defeated mice which showed heightened anxiety

**Figure 3:**
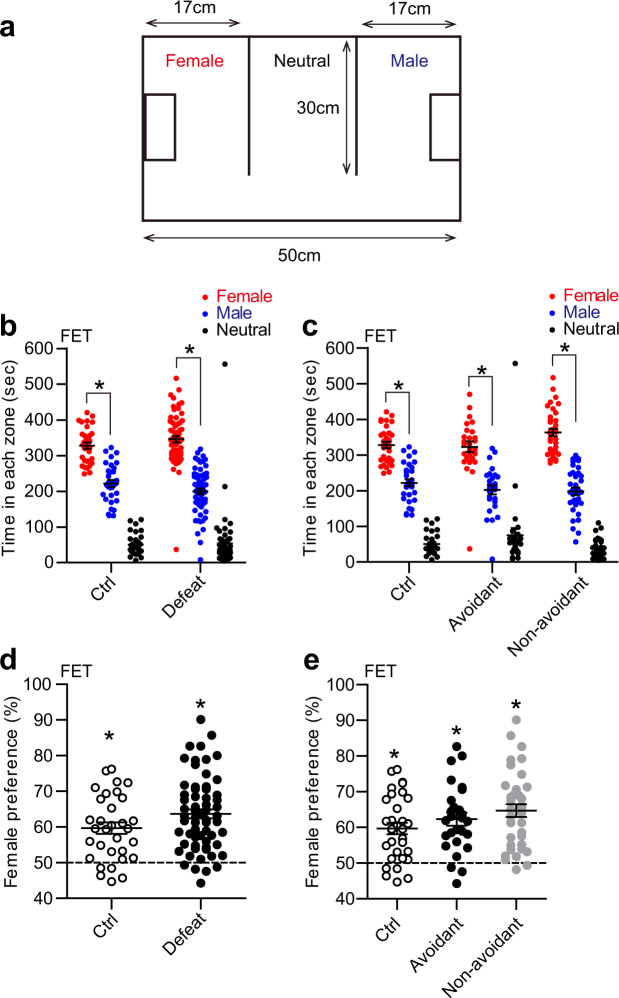
Female preference was indifferent to the level of social avoidance after repeated social defeat stress. (**a**) An experimental chamber used in the female encounter test (FET). The chamber was composed of three interconnected compartments (female zone, male zone and neutral zone). See Materials and Methods for detail. (**b**–**e**) Time spent in each zone (**b**,**c**) and female preference (**d**,**e**) in the female encounter test for control mice (“Ctrl”) and defeated mice (“Defeat”) or for control mice, Avoidant mice and Non-avoidant mice. We categorized the defeated mice that spent more than 50% of the total time in the avoidance zone as the Avoidant mice and the others as the Non-avoidant mice. Values are expressed as means ± SEM. *P < 0.05 for one-way analysis of variance followed by multiple comparison tests with Tukey-Kramer correction (**b**,**c**) and single *t* tests to compare with the chance level (i.e. 50%) (**d**,**e**).

Then we assessed the effects of R-SDS on sexual preference using the female encounter test^9^ (Figs 2a and 3a). In this test, the defeated mice and the control mice were allowed to interact with a novel female mouse and a novel male mouse, each of which was placed on one compartment or the other (“female zone” or “male zone”) of a behavioral chamber (Fig. 3a). These two compartments were interconnected with the third compartment (“neutral zone”). Both the control mice and the defeated mice spent more time in the female zone than in the male zone (Fig. 3b). Female preference was determined by the ratio of the time spent in the female zone to that spent in either the female or male zone. Whereas female preference of both the control mice and the defeated mice was significantly above the chance level (i.e. 50%), no significant difference was found between these two groups of mice (Fig. 3d). Neither the Avoidant mice nor the Non-avoidant mice showed significant difference in female preference from the control mice (Fig. 3c,e).

**Figure 4:**
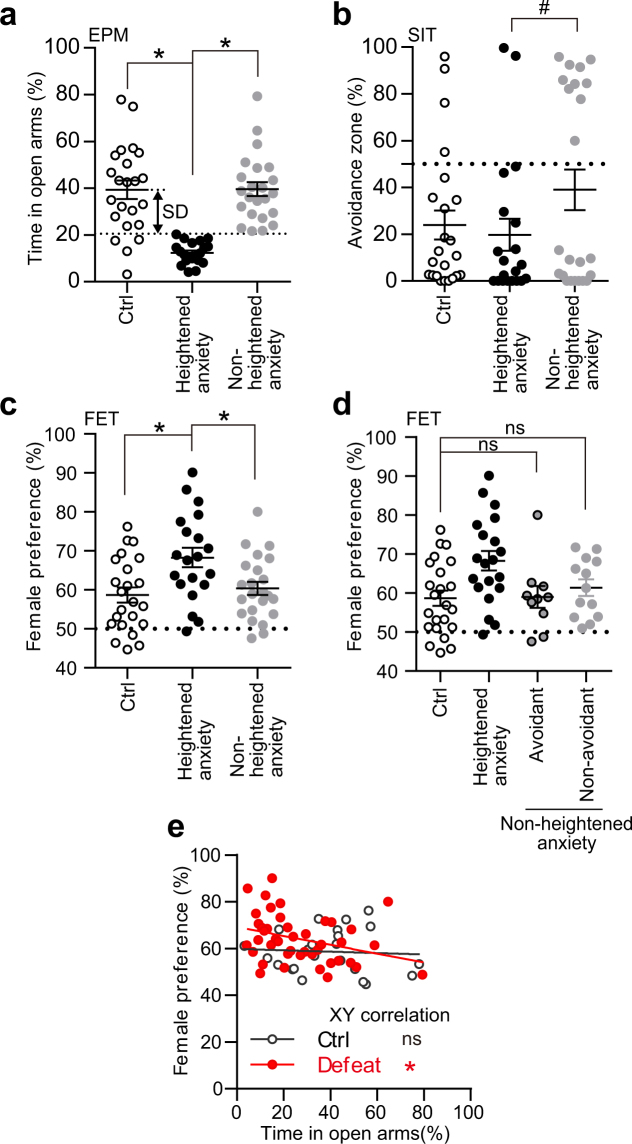
Repeated social defeat stress increased female preference specifically in those with heightened anxiety. (**a**–**d**) The time spent in the open arms in the elevated plus maze test (EPM) (**a**), time spent in the avoidance zone in the social interaction test (SIT) (**b**), and female preference in the female encounter test (FET) (**c**,**d**). The values for control mice (“Ctrl”), the Heightened-anxiety mice and the Non-heightened anxiety mice (**a**–**c**) or those for Ctrl, the Heightened-anxiety mice, the Avoidant mice in the Non-heightened anxiety mice and the Non-avoidance mice in the Non-heightened-anxiety mice (**d**) are shown. See the Results section for the definitions of Heightened-anxiety mice and Non-heightened-anxiety mice. (**e**) Relationship between the time spent in the open arms in the EPM test and female preference in the female encounter test. Values are expressed as means ± SEM. ns, not significant and *P < 0.05 for one-way analysis of variance followed by multiple comparison tests with Tukey-Kramer correction (**a**,**c**,**d**). ^#^*P* < 0.05 for the Fisher’s exact test to compare the proportions of the Avoidant mice and the Non-avoidant mice in the Heightened-anxiety mice and the Non-heightened-anxiety mice (**b**). ns, not significant and *P < 0.05 for the Pearson test for correlation analysis (**e**).

Based on our observation that the effects of R-SDS on the level of anxiety varied among the defeated mice (see Fig. 2f), we sought to categorize the defeated mice according to the level of anxiety and examined whether R-SDS alters female preference in the defeated mice with or without the heightened anxiety. For this purpose, we used the mean minus standard deviation of the time that the control mice spent in open arms of the EPM as the threshold to detect R-SDS-induced heightened anxiety. Indeed, the proportions of the mice below and above the threshold were statistically different between the defeated mice and the control mice (20 mice below the threshold and 23 mice above the threshold for the defeated mice, and 4 mice and 19 mice, respectively, for the control mice; Fisher’s exact test, *P* = 0.0306). Thus, we decided to categorize the defeated mice which showed the time for the open arms below or above the threshold as the Heightened-anxiety mice or the Non-heightened-anxiety mice, respectively. We compared the time in the open arms among the groups of mice, and found that the means are similar between the Non-heightened-anxiety mice (39.6 ± 3.0%) and the control mice (39.3 ± 3.9%), but are significantly different between the Heightened-anxiety mice (12.3 ± 1.0%) and the control mice (39.3 ± 3.9%) (Fig. 4a). Accordingly, one-way analysis of variance followed by Tukey-Kramer multiple comparisons test revealed a significant main effect of Mouse group (*F*(2, 63) = 27.91, *P* < 0,0001) and statistically significant differences across the groups: control vs Heightened-anxiety (*P* < 0.0001, Hedge’s *g* = 1.91), Heightened-anxiety vs Non-heightened-anxiety (*P* < 0.0001, Hedge’s *g* = 2.48), and control vs Non-heightened-anxiety (*P* = 0.883, Hedge’s *g* = 0.02). These results provided statistical rationales for this method to categorize the defeated mice according to the level of anxiety. Consistent with our observation that R-SDS induced the heightened anxiety in the Non-avoidant, but not Avoidant, mice (see Fig. 2f), the proportion of the Avoidant mice was significantly smaller in the Heightened-anxiety mice than in the Non-heightened-anxiety mice (Fig. 4b; 2 Avoidant mice and 18 Non-avoidant mice in the Heightened-anxiety mice, and 10 Avoidant mice and 13 Non-avoidant mice in the Non-heightened-anxiety mice; *P* = 0.0193 by Fisher’s exact test). Notably, the Heightened-anxiety, but not Non-heightened-anxiety, mice showed R-SDS-induced increase in female preference (Fig. 4c). One-way analysis of variance and post hoc Tukey-Kramer multiple comparisons test revealed a significant effect of the Mouse group on female preference (*F*(2,63) = 6.09, *P* = 0.0038) and a significant increase in female preference in the Heightened-anxiety mice, compared with the control mice (*P* = 0.0044, Hedge’s *g* = 0.94) and the Non-heightened-anxiety mice (*P* = 0.0227, Hedge’s *g* = 0.82). No significant difference was found between the control mice and the Non-heightened-anxiety mice (*P* = 0.8176, Hedge’s *g* = 0.19). Female preference in the Avoidant and Non-avoidant subgroups of the Non-heightened anxiety mice was also similar to that in the control mice (Fig. 4d). In addition, female preference was negatively correlated with the time spent in the open arms in the defeated mice (Pearson *r* = −0.3219, *P* = 0.0353), but not in the control mice (Fig. 4e; Pearson *r* = −0.05986, *P* = 0.7861). These findings indicated that R-SDS increases female preference in a manner related to the heightened anxiety, but not to the social avoidance.

## Discussion

In this study, we examined the effects of R-SDS on attentional set shifting and sexual preference in addition to social interaction and anxiety and their relationships. This study provides three novel findings. First, R-SDS impaired attentional set shifting irrespective of the development of social avoidance. This finding showed that attentional set shifting is more sensitive to R-SDS compared with social interaction. Second, R-SDS heightened anxiety in the EPM test only in the defeated mice which did not show social avoidance. Thus, this finding indicated that social avoidance and heightened anxiety can be separately induced by R-SDS at least in our experimental conditions. Third, R-SDS unexpectedly increased the preference to female, one of natural rewards for males examined in R-SDS for the first time, in a subpopulation of the defeated mice which showed heightened anxiety. Female preference after R-SDS was similar irrespective of the development of social avoidance. Taken together, these findings indicated that R-SDS impairs attentional set shifting irrespective of the development of social avoidance and increases female preference associated with heightened anxiety, but not with social avoidance.

The mechanism underlying the difference in stress susceptibility between social interaction and attentional set shifting remains elusive. The medial prefrontal cortex has been implicated in both the impairment in attentional set shifting and the development of social avoidance induced by repeated environmental stress^10,11,17^. It has been reported that the injection of SCH23390, a D1-like antagonist, into the prelimbic area of the medial prefrontal cortex impairs attentional set shifting^18^. By contrast, dopaminergic lesion or knockdown of D1 receptor subtype centered at the infralimbic area of the medial prefrontal cortex facilitates the development of social avoidance by R-SDS^10,11^. Thus, altered dopaminergic activity in different prefrontal areas, namely prelimbic and infralimbic areas, could underlie R-SDS-induced impairment in attentional set shifting and social avoidance, respectively. Whether these prefrontal areas and their dopaminergic activities are differentially affected by R-SDS in a manner related to the behavioral changes remains to be studied.

As mentioned above, R-SDS heightened anxiety only in a subpopulation of the defeated mice which did not show social avoidance. This finding is in contrast to a previous report that R-SDS heightened anxiety irrespective of the development of social avoidance^7^. This discrepancy could be due to subtle differences in experimental conditions in R-SDS (e.g., the duration of each SDS episode, the number of defeated mice simultaneously exposed to an aggressor mouse in each SDS episode, the presence or absence of sensory contact in the intervals between SDS episodes, the presence or absence of brief social isolation prior to R-SDS). It is noticeable that the time spent in the open arms in that study was much lower, and thus the basal level of anxiety was much higher, than in the present study. We speculate that the basal increase in anxiety may allow the defeated mice with social avoidance to show heightened anxiety as well, though this possibility remains to be tested.

R-SDS-induced increase in female preference is contrary to the original expectation based on previous reports that R-SDS as well as other types of repeated stress decreased preference to sucrose, another natural reward^1,2,7,19^. However, this increased female preference is consistent with another report that R-SDS enhanced the motivational drive for sucrose reward in an operant behavior^20^. As most previous studies have focused on stress-induced anhedonia, the concept of stress-induced increase in reward preference has rarely been reported. This finding may provide a novel framework exploitable for understanding stress-related pathology in mental illnesses, such as stress-induced reinstatement of drug addiction after the abstinence.

Notably, we found that R-SDS increases female preference, but not social avoidance, in a subpopulation of the defeated mice which show heightened anxiety. This finding indicated that the level of social avoidance alone cannot recapitulate stress susceptibility of each animal, which is differentially distributed among multiple behaviors. The mechanism about how R-SDS increased anxiety and female preference in the same subpopulation remains elusive. It has been reported that dopamine signaling in nucleus accumbens promotes female preference in rodents, and dopamine and its receptors in nucleus accumbens have been associated with the level of anxiety in rodents and humans^21–23^. Interestingly, it has been reported that suppression of CREB activity in nucleus accumbens impairs R-SDS-induced social avoidance and heightened anxiety as measured by the EPM test^24–26^. Thus, nucleus accumbens could be involved in coordinating the effects of R-SDS on anxiety and sexual preference.

Collectively, the multi-behavioral analyses in R-SDS revealed at least three groups of the defeated mice, namely (1) Heightened-anxiety and Non-avoidant mice, (2) Non-heightened-anxiety and Avoidant mice and (3) Non-heightened-anxiety and Non-avoidant mice, and only the first group of mice showed the increased female preference. These findings indicated that individual variability of stress susceptibility is different between social interaction and anxiety/female preference. Since all the mice used in this study were genetically identical, this individual variability of stress susceptibility could be attributed to environmental factors including rearing and social conditions before R-SDS. According to previous reports, prior social hierarchy is related to the level of social avoidance after R-SDS^27^, and stressors at early life stages affect various behaviors in adulthood^28,29^. Thus, individual variability of stress susceptibility could be different among mice which grew in different environments. Understanding the long-term effects of early-life environmental factors on neural functions in adulthood may help elucidate the biological basis on how each individual animal shows a distinct pattern of stress susceptibilities among multiple behaviors. Since several mental illnesses, such as depression, are more prevalent in females, it remains to be tested whether the patterns of stress-induced behavioral changes in male mice are the same or different in those in female mice.

Since stress susceptibility is associated with the pathologies of mental illnesses, it is important to visualize the behavioral effects of stress in these patients. Among the behaviors tested in this study, behavioral flexibility measured by attentional set shifting is most susceptible to R-SDS. This behavioral test, which can be measured across species including humans^30,31^, may be useful as a sensitive translational probe of stress condition. Furthermore, the patterns of multiple stress-induced behavioral changes found in this study may be exploitable for understanding the heterogeneity of stress-related pathologies among individuals.
